# Comparative Effects of Narrow vs. Wide Cuff Blood Flow Restriction on Muscle Synergy Dynamics: A Time-Frequency Decomposition Approach

**DOI:** 10.3390/s25103154

**Published:** 2025-05-16

**Authors:** Shuai Chang, Chenxi Hu

**Affiliations:** 1 Department of Physical Education, Capital Normal University, No. 105 West Third Ring Road North, Haidian District, Beijing 100048, China; changshuaicnu@163.com; 2 Sport Biomechanics Center, Institute of Artificial Intelligence in Sports, Capital University of Physical Education and Sports, No. 11 North Third Ring Road West, Beijing 100191, China

**Keywords:** surface electromyography, blood flow restriction training, time-frequency analysis, wavelet packet transform, muscle synergy, squat movements

## Abstract

Blood Flow Restriction Training (BFRT) is a training method typically performed with low-intensity loads, yet it has been shown to induce muscle growth and strength gains similar to those achieved through high-load resistance training. This study investigates how different cuff widths affect muscle activation and synergy during squat exercises under BFRT conditions, using wavelet packet transform combined with non-negative matrix factorization (WPT-NNMF) for time-frequency analysis of muscle synergy. Fifteen male participants, each with more than three years of resistance training experience, performed squats under three conditions: non-BFRT (Non-BFRT), BFRT with a 5 cm cuff (5 cm-BFRT), and BFRT with a 10 cm cuff (10 cm-BFRT), all at 30% of their one-repetition maximum (1RM). Surface electromyography (sEMG) signals were recorded from eight lower-limb muscles, and muscle synergy patterns were analyzed using NNMF and WPT-NNMF. The results showed that, compared to Non-BFRT, the 10 cm-BFRT condition significantly increased activation in the vastus lateralis (VL), gluteus maximus (GM), tibialis anterior (TA), and lateral gastrocnemius (GL), while the 5 cm-BFRT decreased activation in the biceps femoris (BF) and increased TA activation. Muscle synergy analysis revealed three distinct synergy modules across all conditions, with the total number of synergies remaining stable. However, the activation weights of muscles within these modules varied across different squat phases, suggesting adaptive neuromuscular regulation under different BFRT conditions. The time-frequency synergy analysis highlighted dynamic changes in muscle coordination across time scales and frequency bands under various training conditions. The number of muscle synergies showed significant changes across different time-frequency regions, with a marked decrease in the 120–250 Hz frequency range in the 5 cm-BFRT condition compared to Non-BFRT. This study is the first to apply time-frequency muscle synergy analysis to investigate the effects of cuff width on neuromuscular coordination during BFRT. The findings offer new insights into the time-frequency characteristics of muscle synergy under BFRT conditions and enhance the understanding of neuromuscular control and motor execution in blood flow restriction training.

## 1. Introduction

Blood Flow Restriction Training (BFRT) is a training method that enhances muscle adaptation by applying external pressure to restrict blood flow to the limbs [1]. This technique utilizes either pneumatic cuffs or elastic bands to partially restrict arterial blood flow while completely occluding venous return, thereby inducing a hypoxic-ischemic state in the distal muscles, which in turn activates metabolic responses and promotes muscle hypertrophy and strength gains [2,3]. BFRT is typically performed at low loads (20–30% 1RM) and for short durations. However, due to the effects of blood flow restriction, muscle fatigue occurs more rapidly, enabling adaptations similar to those achieved through high-intensity training even at lower intensities [4]. The underlying physiological mechanisms of BFRT may involve a venous pooling effect due to restricted blood flow, leading to the accumulation of lactate metabolism [5], mechanotransduction processes [6], and hormonal responses [7], all of which contribute to muscle hypertrophy and strength development [8].

A study has shown that the accumulation of metabolic byproducts during BFRT increases motor unit firing rates and facilitates the recruitment of lower-threshold motor units [9]. Compared to traditional high-intensity resistance training, BFRT performed at low intensities reduces mechanical stress and potential damage to muscles and bones while still eliciting similar adaptations through hormonal stimulation [10]. The hypoxic environment induced by BFRT decreases the recruitment of slow-twitch fibers, leading to a compensatory increase in the activation of fast-twitch fibers to sustain movement execution [11]. Clark et al. compared high-intensity resistance training and low-intensity BFRT, finding that after four weeks of BFRT, both training modalities significantly increased muscle activation and fibrinolytic activity in healthy adults without impairing neural or vascular function [12]. Furthermore, a meta-analysis including 27 studies with 853 participants across different age groups demonstrated that BFRT and traditional high-intensity resistance training resulted in comparable strength gains, with no significant differences between the two methods [13]. Another study indicated that low-intensity BFRT serves as a safe alternative to high-intensity resistance training, significantly enhancing quadriceps strength and muscle cross-sectional area in older adults [14]. Collectively, these findings suggest that low-intensity BFRT elicits muscle activation responses similar to high-intensity resistance training, is applicable to diverse populations, and demonstrates a high level of safety.

In existing BFRT research, cuff widths ranging from 3 to 18 cm have been employed, with 5 cm defined as “narrow”, 10–12 cm as “medium”, and 17–18 cm as “wide” [15]. Under identical percentages of arterial occlusion pressure (AOP), cuff width can markedly alter the degree of blood flow restriction, a factor that must be considered in practical applications. Cuff width modulates restriction by influencing the distribution of occlusion pressure: at the same AOP percentage, a 5 cm cuff requires approximately 147 mmHg to occlude distal upper-arm arteries, whereas a 10 cm cuff achieves equivalent occlusion at around 123 mmHg [16]; similarly, narrower cuffs generally demand higher absolute pressures to match the restriction produced by wider cuffs [17]. These pressure differences not only affect occlusion efficiency but also impact tissue metabolism and training experience: although low-intensity BFRT significantly enhances quadriceps hypertrophy, muscle growth within the cuffed region may be locally attenuated [18]; yet in terms of acute neuromuscular activation, no significant differences in EMG activity of the rectus femoris, vastus lateralis, and vastus medialis have been observed between 5 cm and 11.5 cm cuffs, indicating limited influence of width on instantaneous muscle activation [19]. Furthermore, 12-week lower-limb BFRT interventions at 80% AOP have shown that both 5 cm and 10 cm cuffs yield equivalent increases in one-repetition maximum and quadriceps cross-sectional area, with no significant differences in rating of perceived exertion or pain perception between groups [20]. However, excessively wide cuffs (13.5 cm) significantly elevate pain perception and cardiovascular stress, underscoring the need to balance occlusion efficacy with comfort and safety when selecting cuff width to optimize BFRT prescription [21]. These findings highlight the influence of cuff width on blood flow restriction; however, the underlying motor control mechanisms governing the selection of optimal cuff width in specific training movements and practical applications remain insufficiently explored.

In previous studies examining changes in muscle activation under BFRT, traditional sEMG research primarily focused on the electrical activity of individual muscles, offering a limited perspective on multi-joint coordination in actual movements. In contrast, the muscle synergy theory posits that the central nervous system (CNS) generates movement commands through the coordinated activation of multiple muscles, thereby providing a more comprehensive understanding of complex motor control patterns [22]. This perspective introduces a novel approach to investigating the neuromuscular control mechanisms underlying blood flow restriction squat training with different cuff widths, particularly within the framework of muscle synergy analysis. Non-negative matrix factorization (NNMF) is a commonly used algorithm for analyzing muscle synergies, effectively decomposing synergy vectors and temporal activation patterns to quantify changes in muscle coordination across different task conditions [23]. The frequency-domain characteristics of sEMG signals reflect various physiological aspects of muscle function, as median frequency and conduction velocity are associated with muscle fiber type and cross-sectional area [24,25]. Muscle contraction is regulated by CNS-mediated motor unit firing frequency, and changes in muscle electrical activity frequency often indicate functional modifications within distinct neural pathways [26]. However, traditional muscle synergy analysis has mainly focused on the time-domain features of EMG signals, neglecting the physiological information embedded in the frequency domain, thereby limiting the understanding of the complexity of neuromuscular control [27]. The frequency-domain characteristics of sEMG provide important physiological insights into motor unit recruitment strategies, muscle fiber type distribution, and the development of central fatigue during sustained contractions [28]. Therefore, incorporating frequency-domain information into muscle synergy analysis is crucial for interpreting the neuromuscular adaptations induced by interventions such as BFRT. To overcome the limitations of pure time-domain synergy analysis, wavelet transform (WT) has been introduced to simultaneously capture the time–frequency characteristics of signals. In an early exploration of time–frequency muscle synergy analysis, Frère et al. applied a wavelet-based decomposition combined with non-negative matrix factorization to characterize the upper limb muscle activation strategies in gymnasts [29]. Although WT enables the time–frequency decomposition of non-stationary signals, its resolution is constrained by a fixed time–frequency localization trade-off, which may limit the precise analysis of short-duration, multi-band muscle events.

Wavelet packet transform combined with non-negative matrix factorization (WPT-NNMF) is an effective tool that integrates time-frequency analysis with muscle synergy analysis [27]. Due to its superior frequency resolution in both high- and low-frequency components, wavelet packet transform (WPT) has been widely applied to time-frequency analysis of non-stationary signals [30]. Compared to conventional wavelet transform, WPT provides finer frequency band decomposition, enabling more precise characterization of time-frequency features and enhancing the analysis of both high- and low-frequency components, making it particularly suitable for complex sEMG signals [31]. By integrating NNMF, WPT-NNMF enables the extraction of muscle synergy patterns from the time-frequency domain, uncovering the coordinated activation and temporal variations of different muscle groups. NNMF decomposes the time-frequency representation of sEMG signals into synergy vectors and corresponding temporal activation patterns, thereby mitigating the assumption biases often associated with traditional methods.

Although previous studies have examined the effects of BFRT on strength development and muscle adaptation, research on the impact of cuff width on training outcomes remains limited, particularly regarding its influence on muscle activation patterns. Moreover, most existing investigations have focused on the macroscopic effects of BFRT, with few addressing the underlying neuromuscular control mechanisms or incorporating time–frequency analyses of muscle coordination. The application of muscle synergy analysis under BFRT conditions, particularly in time–frequency domains, remains underexplored. In light of these gaps, the present study aims to investigate how different cuff widths modulate time–frequency muscle synergy patterns during back-squat movements, using a WPT-NNMF framework to reveal potential neuromuscular control adaptations associated with varying compression conditions.

## 2. Methods

### 2.1. Subjects

This study recruited 15 healthy male university students from the Capital University of Physical Education and Sports. The participants had at least three years of resistance training experience, a back squat one-repetition maximum (1RM) exceeding 1.5 times their body weight, and demonstrated proficiency in executing the squat movement with proper technique (age: 23.4 ± 2.2 years; height: 178.5 ± 5.4 cm; weight: 76.7 ± 7.5 kg; 1RM: 139.4 ± 21.8 kg).

All participants were free of musculoskeletal injuries and joint disorders and had no history of cardiovascular disease, hypertension, thrombosis, or varicose veins in the past year. Each participant voluntarily agreed to participate in the study and provided written informed consent before the commencement of the formal testing. The study design and procedures adhered to ethical standards and the principles of the Declaration of Helsinki and were approved by the Ethics Committee of the Capital University of Physical Education and Sports (Approval No. 2025A074).

### 2.2. 1RM Test

Participants first performed a 10 min treadmill warm-up, followed by a warm-up set at 40–60% of their estimated 1RM, completing 5–10 repetitions of the squat exercise. After a 1 min rest, they performed another set at 60–80% of their estimated 1RM for 3–5 repetitions to further activate the muscles and prepare for testing. During the formal 1RM test, participants began with an attempt at 90% of their estimated 1RM. If successful, the load was progressively increased based on participant feedback to approach their maximal capacity. If unsuccessful, the highest successfully lifted load from the previous attempt was recorded as their 1RM. The test typically required 3–5 attempts, with 3–5 min of rest between trials to ensure adequate recovery. The final recorded 1RM was defined as the maximum weight lifted with proper technique, ensuring the squat depth reached 90° of knee flexion [32]. The 1RM test was conducted three days before the BFRT sessions to allow for sufficient recovery.

### 2.3. sEMG Select

This study utilized the 16 wireless surface sEMG (Delsys Inc., Natick, MA, USA) system to measure muscle activity in eight lower-limb muscles: rectus femoris (RF), vastus lateralis (VL), vastus medialis (VM), semitendinosus (ST), biceps femoris (BF), gluteus maximus (GM), tibialis anterior (TA), and gastrocnemius lateral (GL). The sampling frequency was set at 2000 Hz. After completing the warm-up, participants were fitted with the sEMG electrodes in preparation for testing. Before each test, the sEMG system was calibrated and checked to ensure proper functionality and signal accuracy. During the experiment, sEMG signals were recorded in real time throughout the testing sessions.

### 2.4. Training Procedure

**Figure 1:**
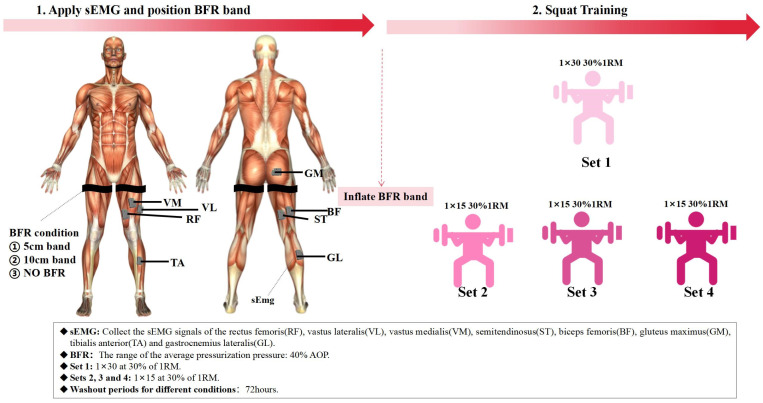
Complete experimental testing process, including the setup for each of the three squat conditions (Non-BFRT, 5 cm-BFRT, and 10 cm-BFRT), the application of blood flow restriction, and the sequence of exercises and measurements performed during the testing sessions.

This study employed a resistance training (squat) protocol using a squat rack (Figure 1). The training protocol consisted of three conditions: back squat without blood flow restriction (Non-BFRT), back squat with 5 cm cuff width BFRT (5 cm-BFRT), and back squat with 10 cm cuff width BFRT (10 cm-BFRT). Each training condition was performed at 30% 1RM and consisted of four sets: the first set included 30 repetitions, while the remaining three sets comprised 15 repetitions each, with a 60 s rest between sets [15]. During BFRT conditions, cuffs were inflated 5 s before the squat exercise using a pneumatic pump. The cuffs were positioned just below the gluteal fold, perpendicular to the longitudinal axis of the thigh [15]. Once the target pressure was reached, inflation was halted, and participants commenced the squatting exercise. At the end of each set, the cuffs were immediately deflated, and re-inflation was initiated 5 s before the next set. This cycle was repeated for all four sets, with the cuffs being completely removed after the final set. The applied pressure was individualized based on the AOP to thigh circumference relationship, as described by Loenneke et al., with all BFRT conditions utilizing 40% AOP [33]. A 72 h interval was maintained between training sessions for each condition.

### 2.5. Data Analysis

#### 2.5.1. Data Pre-Processing

**Figure 2:**
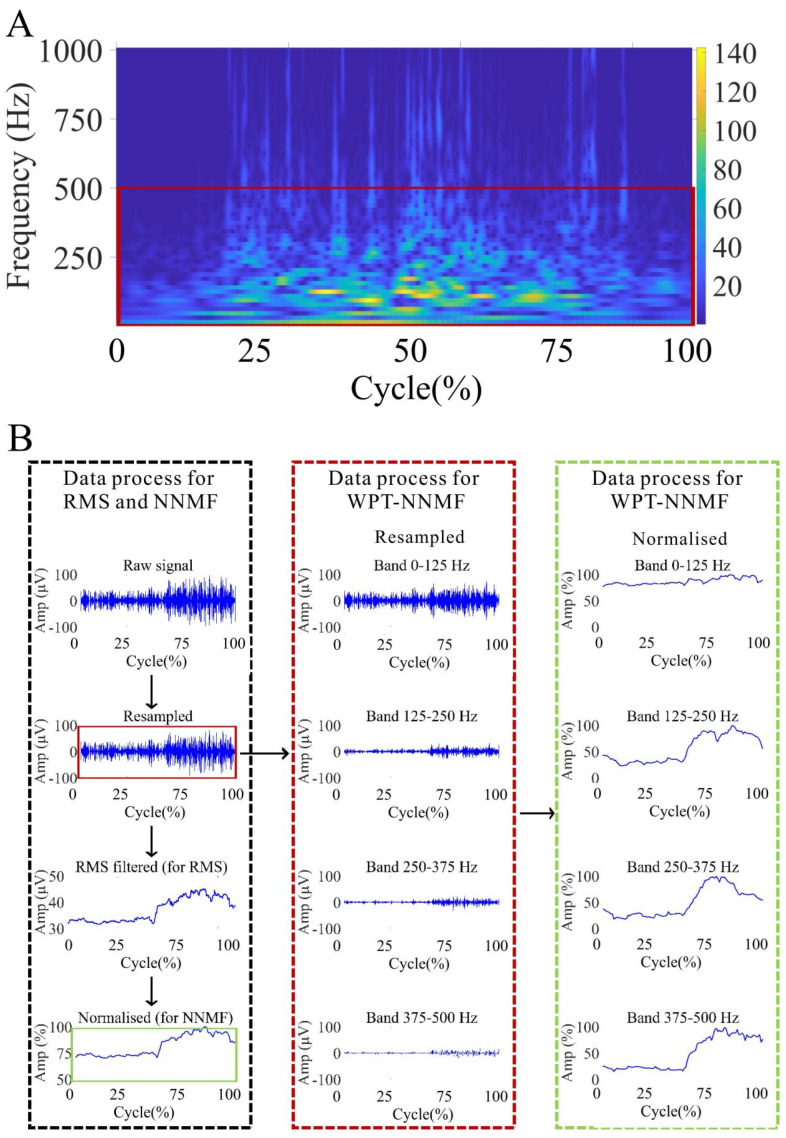
(**A**) Frequency band of interest for the biceps femoris (BF) in a representative subject. The time-frequency distribution, averaged over ten squat repetitions, was obtained using CWT. (**B**) The flow of data preprocessing.

The data were analyzed using NNMF and WPT-NNMF. The sampling frequency of the raw signal was set at 2000 Hz, which is sufficiently higher than the physiological frequencies of muscle activity. Without appropriate frequency band selection, some high-frequency components extracted by WPT-NNMF could lack physiological relevance. Therefore, in the pre-processing stage, it was necessary to determine the effective frequency range for targeted analysis. To achieve this, continuous wavelet transform (CWT) was performed on each raw sEMG signal using MATLAB 2022a, with the “dmey” wavelet selected as the mother wavelet (center frequency = 0.6634 Hz). A two-level decomposition was applied, yielding four sub-bands with a frequency resolution of 125 Hz. The center frequencies of the resulting sub-bands were 62.5 Hz, 187.5 Hz, 312.5 Hz, and 437.5 Hz, respectively. The scalograms revealed that the energy of the sEMG signals in almost all datasets was predominantly concentrated below 500 Hz. This observation informed the selection of the 0–500 Hz frequency band for further analysis. Moreover, the dominance of EMG energy below 500 Hz aligns with known physiological characteristics, as frequencies above this threshold are more likely to represent noise and artifacts rather than meaningful muscle activity [34,35]. Consequently, focusing on the 0–500 Hz band enhances the biological interpretability of the extracted muscle synergies. A representative CWT scalogram from one participant is shown in Figure 2A.

For the NNMF analysis, there are three steps. In step one, all sEMG signals were downsampled to 1000 samples/s for further analysis. In step two, the signals were RMS filtered with a sliding window of length 100 samples to obtain the envelope sEMG signals for RMS and further. In step three, EMG data of each muscle were activation-normalized to its maximum value and time-normalized to 101 points representing 0% to 100% of the overall trial time (Figure 2B). Additionally, the squat onset time for each group was identified using accelerometer data. EMG data from five squat cycles were extracted for each squat movement, with the time of each squat cycle standardized. The average waveform was then calculated from 20 squat cycles to ensure consistency in the analysis.

For the WPT-NNMF analysis, downsampled signals were decomposed into 4 frequency bands by WPT, then the second and third steps mentioned above were carried out (Figure 2B).

#### 2.5.2. NNMF Analysis

In this study, NNMF was employed to perform dimensionality reduction on the sEMG signal matrix (V_mn_), decomposing it into synergy vectors (W_mi_) and time coefficients (C_in_) as follows:

$$V_{mn}\approx \sum_{i=1}^{k}W_{mi}C_{in}=V_{mn}^{\prime }$$

The original sEMG signal matrix $V_{mn}$ consists of m channels and n sampling points.The matrix is decomposed into the muscle synergy vector matrix $W_{mi}$, which indicates the activation weights of each muscle within each synergy module ($W_{mi}$ > 0.3 is used to identify muscles as primary contributors to a specific synergy, and the temporal coefficient matrix $C_{in}$, which represents the time-varying contribution of the i-th synergy module; $V_{mn}^{\prime }$ represents the reconstructed sEMG signal matrix.

(2) The number of synergies k was determined using the Variability Accounted For (VAF) method, with an overall VAF threshold set at 0.9 [36]. The calculation formula for VAF is as follows:

$$VAF=1-\frac{RSS}{TSS}=1-\frac{\sum (V_{mn}-V_{mn}^{\prime }{)}^{2}}{\sum V_{mn}^{2}}$$

where RSS represents the Residual Sum of Squares and TSS represents the Total Sum of Squares.

WPT-NNMF-Based sEMG Signal Processing:

(1) Using wavelet packet transform (WPT), the original signal was decomposed into multiple frequency bands. The signal in each frequency band is represented as follows:

$$x_{i,j}^{N}(t)=\sum_{\tau }X_{i,j,N}^{D}(\tau )\phi_{i,N,\tau }(t)$$

where $x_{i,j}^{N}(t)$ represents the signal in the i-th channel, j-th layer; $X_{i,j,N}^{D}$ denotes the wavelet packet coefficients, representing the projection weights of the signal on the current wavelet basis; $\phi_{i,N,\tau }(t)$ is the basis function of the N-th sub-band, used to capture the local time-frequency features.

(2) Perform NNMF decomposition:

$$V_{j}^{N}\approx W_{j}^{N}C_{j}^{N}$$

where $V_{j}^{N}$ represents the time-frequency signal matrix corresponding to the j-th layer and N-th sub-band. $W_{j}^{N}$ represents the synergy matrix, characterizing the features of different synergy patterns in the sub-band signal.$C_{j}^{N}$ represents the temporal coefficient matrix, indicating the time-varying contributions of the synergy patterns.

#### 2.5.3. Outcome Variables

The primary independent variables were cuff condition (Non-BFRT, 5 cm-BFRT, 10 cm-BFRT), lower-limb muscle (RF, VL, VM, ST, BF, GM, TA, GL), NNMF-derived synergy module (Module 1, Module 2, Module 3), and frequency band for WPT-NNMF (0–125 Hz, 125–250 Hz, 250–375 Hz, 375–500 Hz). The dependent outcome measures comprised the following: (1) RMS amplitude, reflecting overall muscle activation; (2) NNMF-derived synergy weights (W), quantifying each muscle’s contribution within its module; (3) the number of modules extracted by NNMF across the three cuff conditions; and (4) the number of modules per cuff per frequency band from WPT-NNMF, indicating the time-frequency feature of muscle synergies (Table 1).

#### 2.5.4. Statistical Analyses

Statistical analyses were conducted using SPSS 25 (IBM, Armonk, NY, USA), with data visualization performed in GraphPad Prism 9 (GraphPad Software, San Diego, CA, USA). WPT-NNMF computations were carried out in MATLAB 2022a (MathWorks, Natick, MA, USA). The Shapiro–Wilk test was first used to assess the normality of the data distribution. For normally distributed data, a repeated-measures ANOVA was performed to examine the effects of different BFRT conditions on outcome variables; Mauchly’s test of sphericity was applied to verify the equality of variances of the differences, and Greenhouse–Geisser corrections were employed whenever sphericity was violated. If a significant effect was detected, paired *t*-tests with Bonferroni–Holm correction were applied for multiple comparisons. For non-normally distributed data, the Friedman test was employed for non-parametric analysis; when significant, the Wilcoxon signed-rank test was conducted as a post hoc test with Bonferroni–Holm correction to control for multiple comparison errors. A two-tailed *p*-value < 0.05 was considered statistically significant.

## 3. Results

### 3.1. RMS Analysis of sEMG Signals During Non-BFRT, 5 cm-BFRT, and 10 cm-BFRT Squats

**Figure 3:**
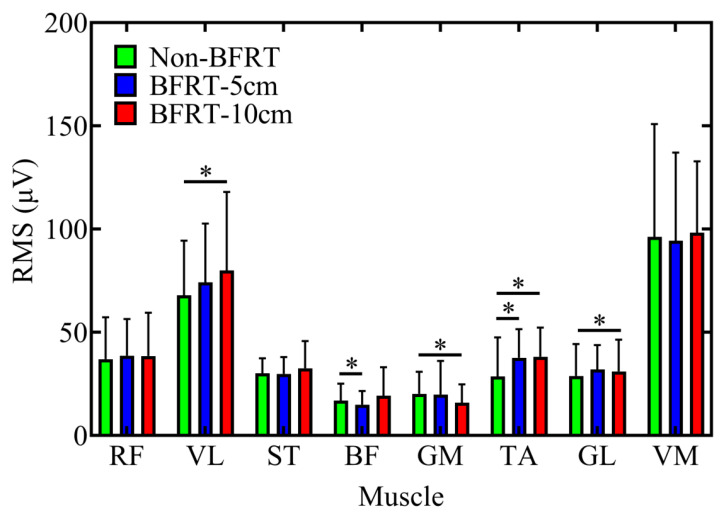
RMS results of sEMG signals during BFRT, where green, blue, and red represent Non-BFRT, 5 cm-BFRT, and 10 cm-BFRT, respectively, for the RMS values of eight muscles. Abbreviations: RF: rectus femoris, VL: vastus lateralis, ST: semitendinosus, BF: biceps femoris, GM: gluteus maximus, TA: tibialis anterior, GL: gastrocnemius lateralis, VM: vastus medialis. * indicates statistically significant difference between conditions (*p* < 0.05).

Significant differences in RMS values of sEMG signals were observed in VL, BF, GM, TA, and GL (Figure 3). Compared to Non-BFRT, VL activation increased in BFRT-10 cm (67.91 ± 26.48 vs. 79.86 ± 38.11; *p* = 0.046). For BF, the BFRT-5 cm condition showed significantly lower values than Non-BFRT (14.91 ± 6.67 vs. 11.76 ± 6.34; *p* = 0.017). In GM, activation decreased in BFRT-10 cm compared to Non-BFRT (20.17 ± 10.71 vs. 15.91 ± 8.88; *p* < 0.001). In TA, activation increased in both BFRT-5 cm and BFRT-10 cm compared to Non-BFRT (28.61 ± 18.96 vs. 37.63 ± 13.86, 28.61 ± 18.96 vs. 38.11 ± 14.18; *p* = 0.025, 0.011). In GL, activation was significantly higher in BFRT-10 cm compared to Non-BFRT (28.81 ± 15.48 vs. 31.01 ± 15.42; *p* < 0.001).

### 3.2. Muscle Synergy Number in BFRT Squats with Different Cuff Widths

**Figure 4:**
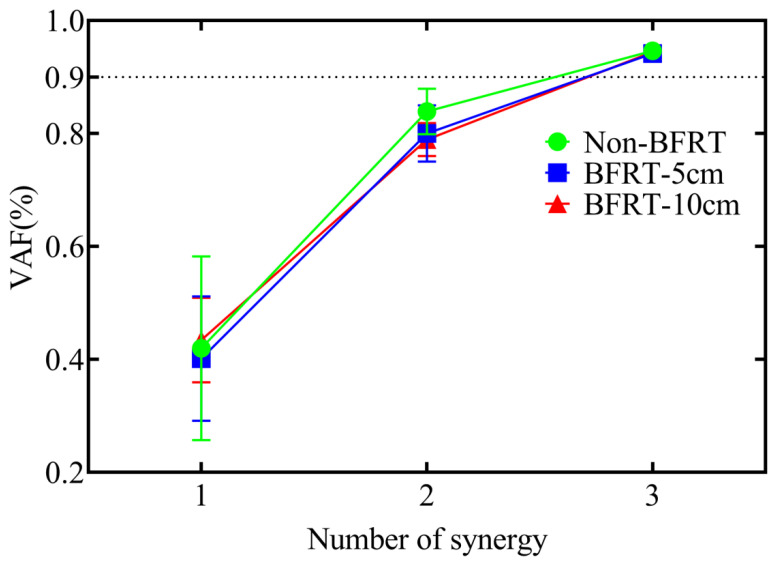
Muscle synergy numbers under three BFRT conditions.

In Non-BFRT, BFRT-5 cm, and BFRT-10 cm squat conditions, three synergy modules were extracted using NNMF. The Friedman test indicated that there were no significant differences in the number of synergy modules among the three conditions(Figure 4).

### 3.3. NNMF Results During BFRT Squats with Different Cuff Widths

Muscles with an activation weight > 0.3 were considered primary contributors to a given synergy [37]. Synergy 1 corresponded to the descending phase of the squat, with RF, ST, and TA playing dominant roles. This synergy was primarily active during the first 30% of the squat phase, contributing to accelerating dorsiflexion in the downward movement. Synergy 2 corresponded to the early ascent phase, dominated by RF, VL, and VM. This synergy was active during approximately 50% of the squat phase, facilitating knee extension acceleration. Synergy 3 corresponded to the late ascent phase, where it remained active for approximately 80% of the squat phase, continuing to support knee extension execution. ST, BF, GM, and GL were the primary contributors to this synergy. Aligned raw EMG traces, cross-participant synergy-activation curves with muscle weights, and the movement schematic are presented in Supplementary Figure S1.

**Figure 5:**
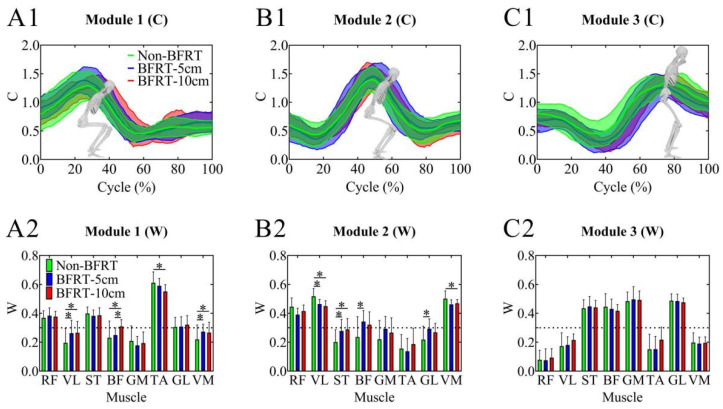
Synergy modules in squats under different BFRT conditions based on NNMF. (**A1**–**C1**) Activation coefficients of the three synergy modules. (**A2**–**C2**) Muscle synergy vectors of the three modules. * Significant differences between groups were identified using paired *t*-tests with Bonferroni–Holm correction following repeated-measures ANOVA (*p* < 0.05).

For Synergy 1, VL activation showed a significant increase in both BFRT-5 cm and BFRT-10 cm compared to Non-BFRT (0.19 ± 0.10 vs. 0.26 ± 0.09, 0.19 ± 0.10 vs. 0.27 ± 0.08; *p* < 0.001, *p* = 0.001). BF activation significantly increased in BFRT-10 cm compared to Non-BFRT (0.23 ± 0.12 vs. 0.31 ± 0.05; *p* = 0.045) and also showed a significant increase in BFRT-10 cm compared to BFRT-5 cm (0.25 ± 0.05 vs. 0.31 ± 0.05; *p* = 0.012). TA activation significantly decreased in BFRT-10 cm compared to Non-BFRT (0.61 ± 0.07 vs. 0.55 ± 0.05; *p* = 0.046). VM activation was significantly higher in both BFRT-5 cm and BFRT-10 cm compared to Non-BFRT (0.22 ± 0.10 vs. 0.27 ± 0.05, 0.22 ± 0.10 vs. 0.27 ± 0.07; *p* = 0.003, *p* = 0.01). For Synergy 2, VL activation significantly decreased in both BFRT-5 cm and BFRT-10 cm compared to Non-BFRT (0.52 ± 0.05 vs. 0.46 ± 0.03, 0.52 ± 0.05 vs. 0.45 ± 0.03; *p* = 0.011, *p* = 0.004). ST activation significantly increased in BFRT-5 cm and BFRT-10 cm compared to Non-BFRT (0.20 ± 0.09 vs. 0.28 ± 0.08, 0.20 ± 0.09 vs. 0.29 ± 0.07; *p* = 0.019, *p* = 0.008). BF activation was significantly higher in BFRT-5 cm compared to Non-BFRT (0.24 ± 0.14 vs. 0.34 ± 0.08; *p* = 0.04). GL activation significantly increased in BFRT-5 cm compared to Non-BFRT (0.22 ± 0.09 vs. 0.29 ± 0.07; *p* = 0.029). VM activation significantly decreased in BFRT-10 cm compared to Non-BFRT (0.50 ± 0.05 vs. 0.46 ± 0.03; *p* = 0.049) (Figure 5).

### 3.4. Muscle Synergy Analysis Within WPT-NNMF

**Figure 6:**
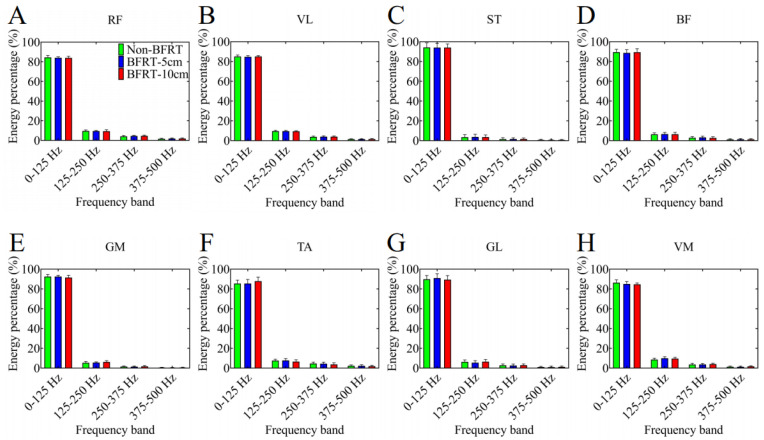
The energy percentage of each muscle in different frequency bands. Subfigures show results for: (**A**) RF—rectus femoris, (**B**) VL—vastus lateralis, (**C**) ST—semitendinosus, (**D**) BF—biceps femoris, (**E**) GM—gluteus maximus, (**F**) TA—tibialis anterior, (**G**) GL—gastrocnemius lateralis, and (**H**) VM—vastus medialis.

After the two-layer decomposition, the four frequency bands from low to high are 0–125 Hz, 125–250 Hz, 250–375 Hz, and 375–500 Hz, respectively (Figure 6). It is evident that as the frequency bands increase, the percentage of energy exhibits a declining trend.

**Figure 7:**
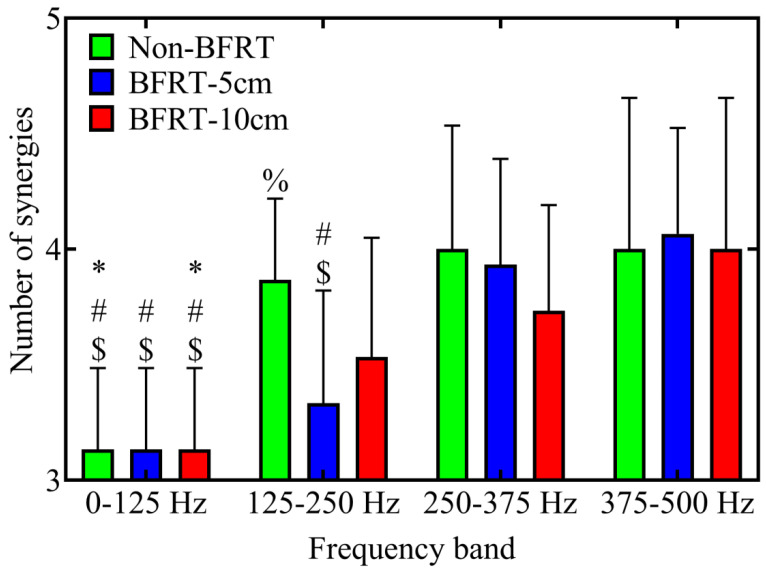
WPT-NNMF analysis of muscle synergy numbers across different frequency bands during squats under Non-BFRT, BFRT-5 cm, and BFRT-10 cm conditions. The number of synergy modules in different tasks and different frequency bands was compared, and the Bonferroni method was used for post hoc multiple comparisons. Salient markers in the figure: *, #, and $ indicate *p* < 0.05 compared to the 125–250 Hz, 250–375 Hz, and 375–500 Hz groups, respectively. % indicates *p* < 0.05 compared to BFRT-5 cm.

Compared to the 0–125 Hz frequency band, the number of muscle synergies significantly increased in Non-BFRT at 125–250 Hz (3.13 ± 0.35 vs. 3.87 ± 0.35, *p* < 0.001), 250–375 Hz (3.13 ± 0.35 vs. 4.00 ± 0.53, *p* < 0.001), and 375–500 Hz (3.13 ± 0.35 vs. 4.00 ± 0.65, *p* < 0.001). Similarly, in BFRT-10 cm, muscle synergy numbers were significantly higher in the 125–250 Hz (3.13 ± 0.35 vs. 3.53 ± 0.52, *p* = 0.047), 250–375 Hz (3.13 ± 0.35 vs. 3.73 ± 0.46, *p* = 0.001), and 375–500 Hz (3.13 ± 0.35 vs. 4.00 ± 0.65, *p* < 0.001) frequency bands. Under BFRT-5 cm, the number of synergies was significantly higher in the 250–375 Hz (3.93 ± 0.46 vs. 3.13 ± 0.35, *p* < 0.001) and 375–500 Hz (4.00 ± 0.65 vs. 3.13 ± 0.35, *p* < 0.001) frequency bands compared to 0–125 Hz. Additionally, within the 125–250 Hz frequency band, BFRT-5 cm exhibited a significantly lower number of synergies than Non-BFRT (3.86 ± 0.35 vs. 3.33 ± 0.49, *p* = 0.008) (Figure 7).

Supplementary Figures S2–S5 presents the individualized WPT-NNMF analysis results for each participant under different frequency bands and BFRT conditions. This visualization intuitively reflects the variations in time-frequency muscle activation patterns across individuals, further confirming the statistical findings and highlighting the presence of inter-individual differences.

## 4. Discussion

This study is the first to use the WPT-NNMF method to analyze the time-frequency muscle synergy patterns during lower limb blood flow restriction squats under different cuff width conditions. RMS analysis showed significant differences in muscle activation, with increased VL and GL activation in 10 cm-BFRT and decreased BF activation in 5 cm-BFRT. Despite these variations in activation, the number of muscle synergies remained stable across all conditions. NNMF results revealed that the activation weights of synergy modules 1 and 2 showed differences under different BFRT conditions, with muscles such as VL, BF, and TA playing a dominant role. Additionally, WPT-NNMF analysis further highlighted differences in muscle synergy numbers across frequency bands, with a significant decrease in synergy numbers in the 125–250 Hz band under the 5 cm-BFRT condition compared to Non-BFRT. This study demonstrates that dynamic squat movements under different blood flow restriction conditions are associated with specific neural inputs, and that motor execution across frequency bands is influenced by both central nervous system regulation and metabolic stress induced by blood flow restriction.

### 4.1. The RMS Results of Squat Movements Under Different Blood Flow Restriction Conditions

Compared to Non-BFRT squats, BFRT-10 cm increased muscle activation in VL, GM, TA, and GL, while BFRT-5 cm reduced BF activation but increased TA activation. The increased activation of VL, GM, TA, and GL under BFRT-10 cm aligns with previous findings, suggesting that BFRT significantly enhances lower-limb muscle activation by restricting blood flow and increasing metabolic demand [38]. VL and GM, as major muscle groups, play a crucial role in squat performance [39], and wider cuffs induce greater blood flow restriction than narrow cuffs [21], potentially enhancing force output and muscular endurance in these key muscles. During squatting, TA and GL serve as stabilizers of the lower leg and support the ankle joint [40]. The increased activation of these muscles may compensate for the elevated load and stability demands imposed by BFR squats.

### 4.2. Changes in Muscle Synergy Numbers Based on NNMF

In this study, although muscle activation intensity varied under different training conditions, the number of muscle synergies remained stable. This stability is closely related to the closed-chain nature of the squat movement and the modular control of the CNS [41]. The CNS coordinates muscle groups in multi-joint movements, ensuring the stability and efficiency of movement patterns. Even when external training loads change, the CNS can adjust muscle activation intensity to maintain motor control strategies without altering muscle synergy numbers. Similar studies have shown that in other motor tasks, the number of muscle synergies remains unchanged under conditions of external load variations or internal fatigue [42,43].

### 4.3. Changes in Muscle Synergy Modules Based on NNMF

In muscle synergy analysis, Synergy 1 occurs during the descending phase of the squat, primarily driven by the hamstrings (ST, BF) and TA, which are responsible for knee flexion and dorsiflexion. Differences in muscle activation were observed under different BFR conditions. Compared to Non-BFRT, VL and VM activation significantly increased under both BFRT-5 cm and BFRT-10 cm conditions. During knee flexion, the quadriceps play a crucial role in maintaining movement stability [44]. BFRT may enhance quadriceps activation during the descending phase by increasing local metabolic demand. Additionally, BF activation significantly increased under BFRT-10 cm, whereas TA activation significantly decreased. Changes in external conditions may lead to compensatory muscle activation responses [45]. BFRT not only stimulates proximal target muscles but also affects distal muscle strength development [46]. The reduced TA activation may result from load redistribution and local blood flow restriction, suggesting that BFRT may alter the activation patterns of smaller muscles during squatting.

Synergy 2 occurs during the early ascent phase, primarily driven by the quadriceps to extend the knee joint. In this study, at the initial stance phase, VL and VM activation decreased under both BFRT-5 cm and BFRT-10 cm compared to Non-BFRT, while ST, BF, and GL activation increased. The quadriceps are primarily responsible for knee stabilization and extension during squats, particularly in the early ascent phase, where they provide substantial force to maintain knee stability [47]. However, our findings revealed a significant reduction in VL activation under BFRT compared to Non-BFRT, possibly due to altered quadriceps activation patterns under BFRT. Previous studies suggest that muscle growth beneath the cuff may be inhibited in BFRT [48]. By restricting blood flow, BFRT increases local metabolic stress, inducing load redistribution and neural adaptations that reduce quadriceps workload. Meanwhile, the enhanced activation of ST, BF, and GL suggests a compensatory mechanism to maintain movement efficiency. These findings highlight the neuromuscular system’s adaptive regulation under acute BFRT, optimizing muscle synergy for training efficacy.

### 4.4. Changes in Muscle Synergy Numbers Across Frequency Bands Under Different Training Conditions Based on Time-Frequency Synergy Analysis

This study examined the effects of BFRT with different cuff widths on muscle synergy numbers and compared neuromuscular synergy patterns across different frequency bands (0–125 Hz, 125–250 Hz, 250–375 Hz, 375–500 Hz) using WPT-NNMF. The results demonstrated a clear frequency-dependent variation in muscle synergy numbers, which were also influenced by BFRT conditions.

Compared to Non-BFRT, BFRT-5 cm significantly reduced muscle synergy numbers in the 125–250 Hz frequency band. Although no direct studies have examined the effects of cuff width on muscle synergy within this frequency range, previous research indicates that BFRT alters the spectral characteristics of sEMG signals and affects motor unit recruitment patterns [49]. Additionally, low-intensity BFRT has been shown to induce neuromuscular adaptations in specific frequency bands [50]. These findings suggest that BFRT may regulate motor unit firing patterns, thereby influencing high-frequency muscle synergy numbers. Moreover, cuff width affects the physiological responses to BFRT, as narrow cuffs require a higher absolute pressure to achieve the same AOP, potentially leading to greater vascular mechanical load and localized tissue compression [51,52]. Narrower cuffs are also associated with greater pain perception and an increased risk of soft tissue damage [53]. Compared to BFRT-10 cm, the smaller compression area of BFRT-5 cm results in more localized blood flow restriction and greater physiological stress, which may alter motor unit recruitment and synergy patterns within this frequency range during squatting.

Under BFRT-5 cm, BFRT-10 cm, and Non-BFRT conditions, muscle synergy numbers in the 250–375 Hz and 375–500 Hz frequency bands increased, though no statistically significant differences were observed among the three conditions. This suggests that high-frequency muscle synergy changes are primarily regulated by neuromuscular control mechanisms rather than being driven by local metabolic stress. Motor unit recruitment patterns exhibit high plasticity across different motor tasks and are modulated by neural input strategies [54]. Furthermore, different motor tasks can induce adjustments in motor unit recruitment strategies, which are predominantly governed by cortical-spinal pathway regulation [22]. Additionally, corticomuscular coherence (CMC), a key component of the neural control system, may play a crucial role in high-frequency muscle synergy regulation. CMC exhibits task-dependent modulation patterns, which are mainly influenced by cortical-spinal signaling [55,56]. The lack of a significant BFRT effect on high-frequency muscle synergy numbers in this study may be due to the longer adaptation period required for CMC, as short-term training may not induce substantial cortical-spinal pathway remodeling.

Although group-level statistical analysis revealed changes in muscle synergy numbers across different frequency bands and training conditions, individual-level analysis demonstrated distinct personalized variations in time-frequency muscle synergy patterns. This suggests that the CNS may exhibit individual adaptability in motor unit control mechanisms, with regulatory strategies potentially influenced by training experience, neural adaptability, and individual differences in muscle fiber composition. These findings further support the hypothesis that the CNS regulates motor tasks through a modular control mechanism [27] and provide insights into how BFRT influences neuromuscular control strategies during squatting. The WPT-NNMF framework applied in this study first decomposes sEMG signals into finely resolved frequency bands and then extracts synergy modules within each band, preserving both temporal activation sequences and spectral detail during blood flow restriction squat movements. In contrast, conventional time-domain NNMF captures only spatial-temporal structures while overlooking frequency content; continuous wavelet and Fourier analyses, while revealing global spectral patterns, cannot isolate modular structures; and traditional EMG metrics such as RMS and median frequency report only overall summaries. By quantifying changes in synergy number and muscle weighting across distinct frequency bands, WPT-NNMF clearly reveals how cuff width differentially modulates neuromuscular synergy within each band, offering deeper insights into BFRT-induced time–frequency coordination dynamics. The observed changes in synergy-module weights and in time–frequency synergy counts across cuff conditions indicate that the central nervous system adjusts intermuscular coordination in response to different compression widths. In this context, our findings show how cuff-width-dependent shifts in muscle activation timing, module weighting, and frequency-band synergy numbers can serve as indirect evidence of adaptive neuromuscular control strategies during BFRT squats.

### 4.5. Muscle Fatigue Considerations in BFRT

This study focused on the time–frequency reorganization of lower limb muscle synergies during squatting under different cuff widths, without concurrent analysis of muscle fatigue indicators. Muscle fatigue is one of the physiological effects induced by BFRT, which is associated with muscular hypertrophy and neural adaptation. Previous studies have shown that narrower cuffs require higher inflation pressures to achieve equivalent arterial occlusion, potentially accelerating metabolite accumulation, intensifying fatigue, and altering motor unit recruitment patterns [57,58]. Therefore, the conclusions regarding cuff width–related differences in neuromuscular synergy should be interpreted with caution, considering the unmeasured fatigue as a potential confounding factor. Future work using the WPT-NNMF framework for time–frequency synergy analysis should incorporate both subjective and objective indicators of fatigue to quantitatively assess fatigue accumulation and evaluate whether it may influence synergy characteristics and the underlying mechanisms.

### 4.6. Limitations

This study represents a preliminary exploration of the time-frequency synergy characteristics of BFRT under different cuff widths, yet certain limitations remain. The sEMG data collection was limited to eight lower-limb muscles, potentially overlooking the contributions of key synergists or antagonists in the trunk, which may result in an incomplete analysis of neuromuscular control strategies. The limited number of muscle channels might have underestimated the true number or spatiotemporal characteristics of synergy modules [59]. Additionally, our relatively small sample size of homogenous, resistance-trained young males further limits the generalizability of these findings to other populations. Furthermore, this study lacked synchronized kinematic and kinetic data, making it difficult to establish causal relationships between muscle synergy patterns and movement mechanics. Moreover, relying solely on sEMG analysis does not directly reveal how BFRT affects CNS control strategies.

Future studies should expand both the number and anatomical distribution of EMG channels to include additional key muscle groups. They should also increase sample size and include more diverse populations to improve external validity. Combining EEG recordings with corticomuscular coherence analysis will enable quantification of corticospinal adaptations to BFRT. Finally, fatigue metrics derived from EMG signals and perceptual measures should be integrated into a comprehensive time–frequency synergy framework to assess how fatigue accumulation affects neuromuscular coordination during BFRT.

## 5. Conclusions

RMS analysis showed that BFRT-10 cm increased VL, GM, TA, and GL activation, indicating that BFRT enhances lower-limb muscle activation by elevating metabolic load. In contrast, BFRT-5 cm led to a decrease in BF activation, likely due to differences in muscle recruitment influenced by cuff width. Muscle synergy analysis demonstrated that synergy numbers remained stable across training conditions, reflecting the CNS’s control stability in closed-chain movements like squatting. NNMF results indicated that during the descending phase, quadriceps activation significantly increased, while during the early ascent phase, ST, BF, and GL activation increased, suggesting that BFRT optimizes CNS-driven compensatory activation strategies. WPT-NNMF analysis further revealed that muscle synergy distribution across frequency bands remained stable, with the 125–250 Hz range being affected by BFRT conditions. These findings provide electrophysiological insights for developing individualized BFRT protocols based on cuff width and expand the research perspective of BFRT related to nervous system motor control.

## Figures and Tables

**Table 1:** Experimental factors, levels, and analytical purposes for muscle activation and synergy measures under blood flow restriction training.

Factor Type	Factor	Levels	Purpose
Independent	Cuff condition	Non-BFRT, 5 cm-BFRT, 10 cm-BFRT	To determine how cuff width modulates muscle activation and synergy patterns
Independent	Muscle	RF, VL, VM, ST, BF, GM, TA, GL	To compare activation (RMS) and synergy contributions (W) across eight lower-limb muscles
Independent	Synergy module (NNMF)	Module 1, Module 2, Module 3	To examine changes in each module’s muscle weights (W) under different cuff conditions
Independent	Frequency band (WPT-NNMF)	0–125 Hz, 125–250 Hz, 250–375 Hz, 375–500 Hz	To assess how time-frequency decomposition influences the number of synergies (K)
Dependent	RMS value	–	Reflects overall muscle activation
Dependent	Synergy weight (W)	–	NNMF-derived contribution of each muscle to its module
Dependent	Synergy module number (NNMF)	–	Number of NNMF modules needed to reach VAF > 0.9 in each frequency band
Dependent	Synergy module number (WPT-NNMF)	–	Number of WPT-NNMF modules extracted (time-domain synergies)

## Data Availability

The raw data supporting the conclusions of this article will be made available by the authors on request.

## Supplementary Materials

The following supporting information can be downloaded at: https://www.mdpi.com/article/10.3390/s25103154/s1.
