# Supplementary figures and images for: Comparative Effects of Narrow vs. Wide Cuff Blood Flow Restriction on Muscle Synergy Dynamics: A Time-Frequency Decomposition Approach
Source: Sensors (Basel). 2025 May 16;25(10):3154. doi: 10.3390/s25103154 (PMC12116078; doi:10.3390/s25103154)

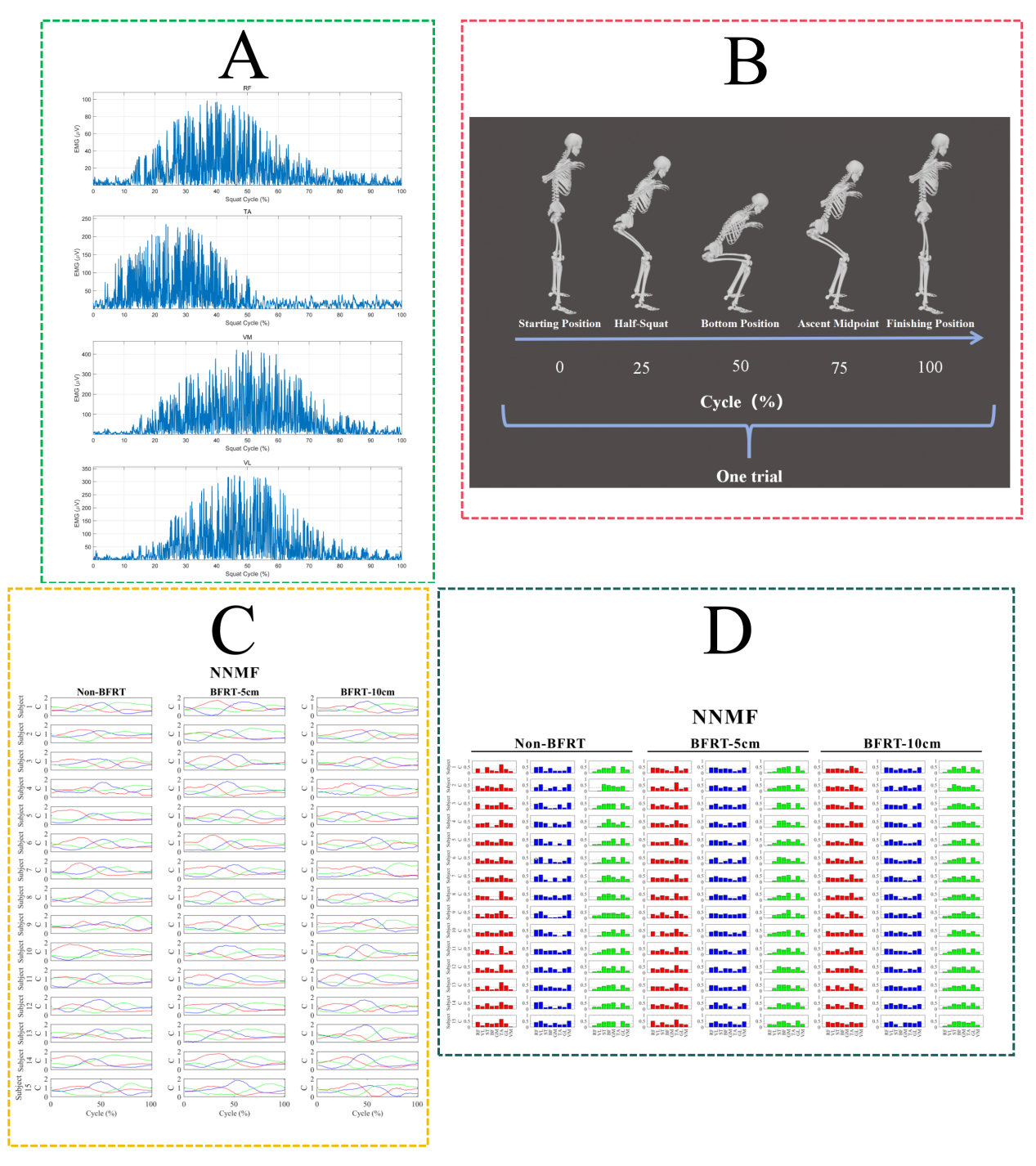

Supplement: Supplementary file 1 [file sensors-25-03154-s001.zip › Supplementary Figure S1.png]

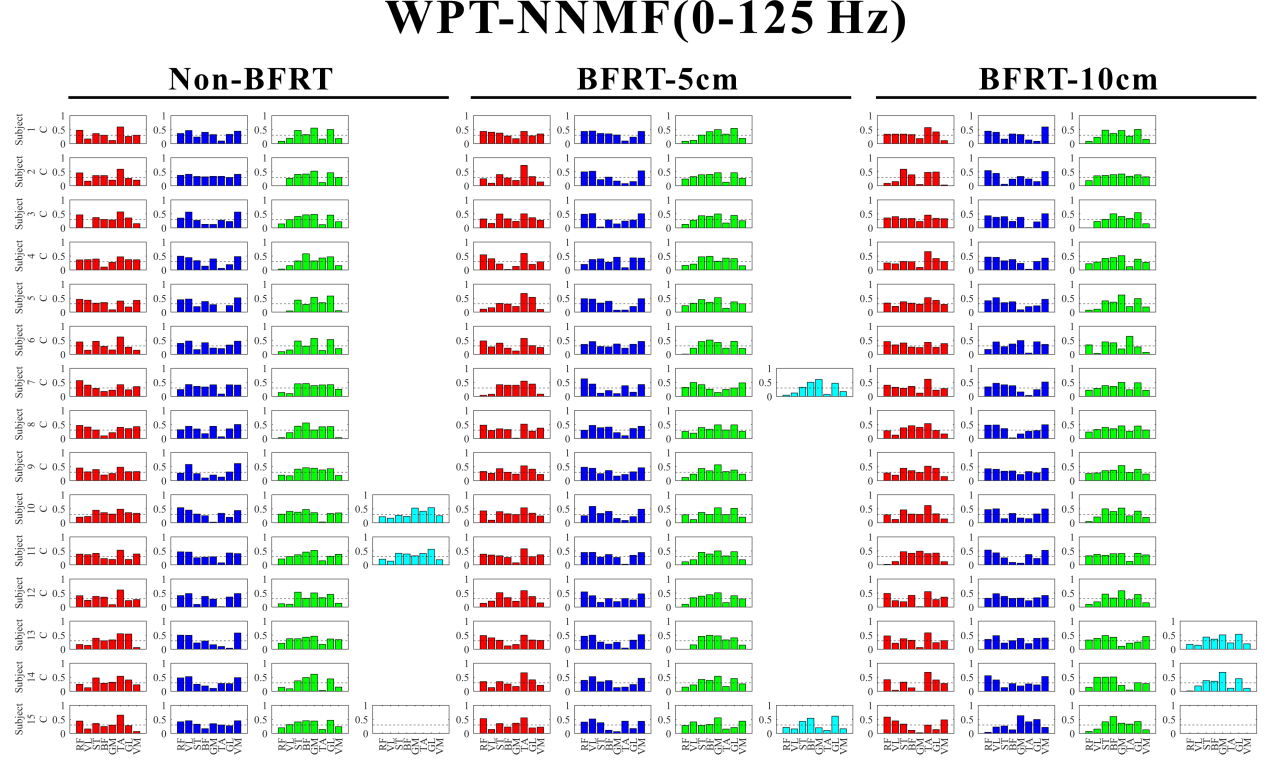

Supplement: Supplementary file 1 [file sensors-25-03154-s001.zip › Supplementary Figure S2.png]

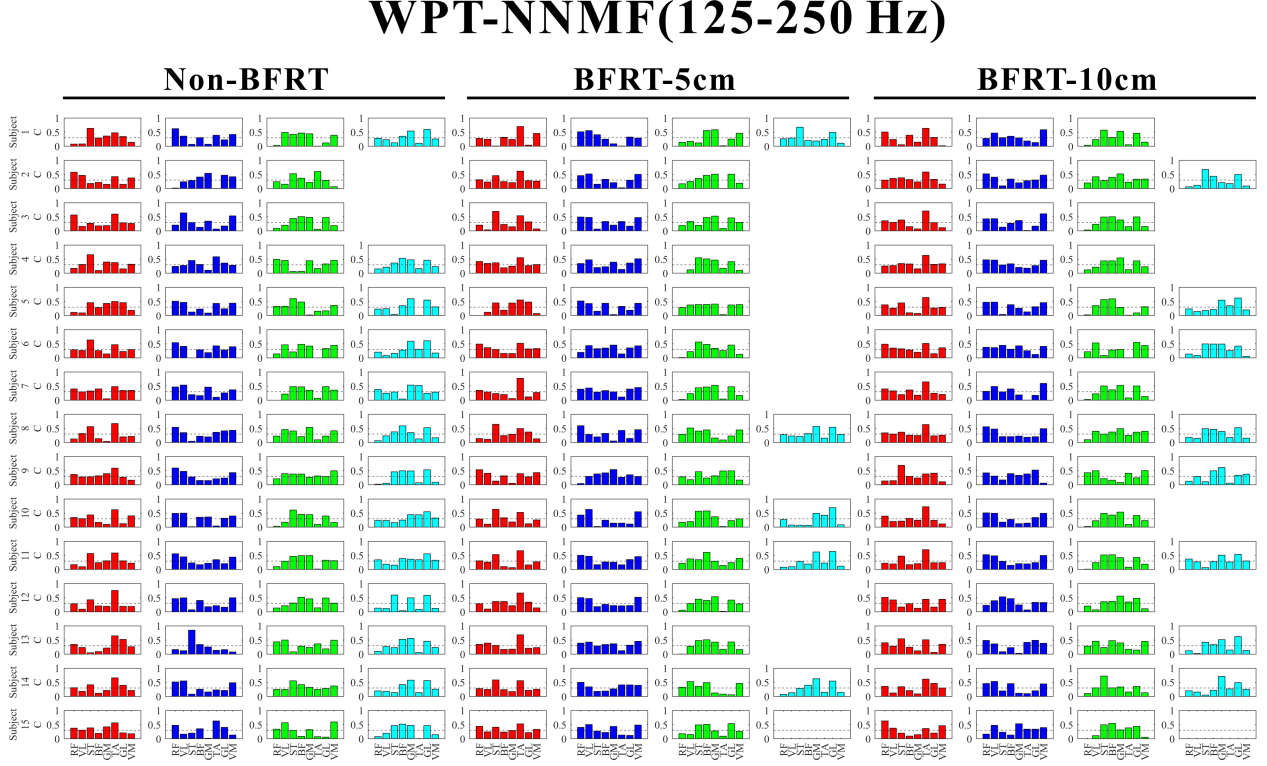

Supplement: Supplementary file 1 [file sensors-25-03154-s001.zip › Supplementary Figure S3.png]

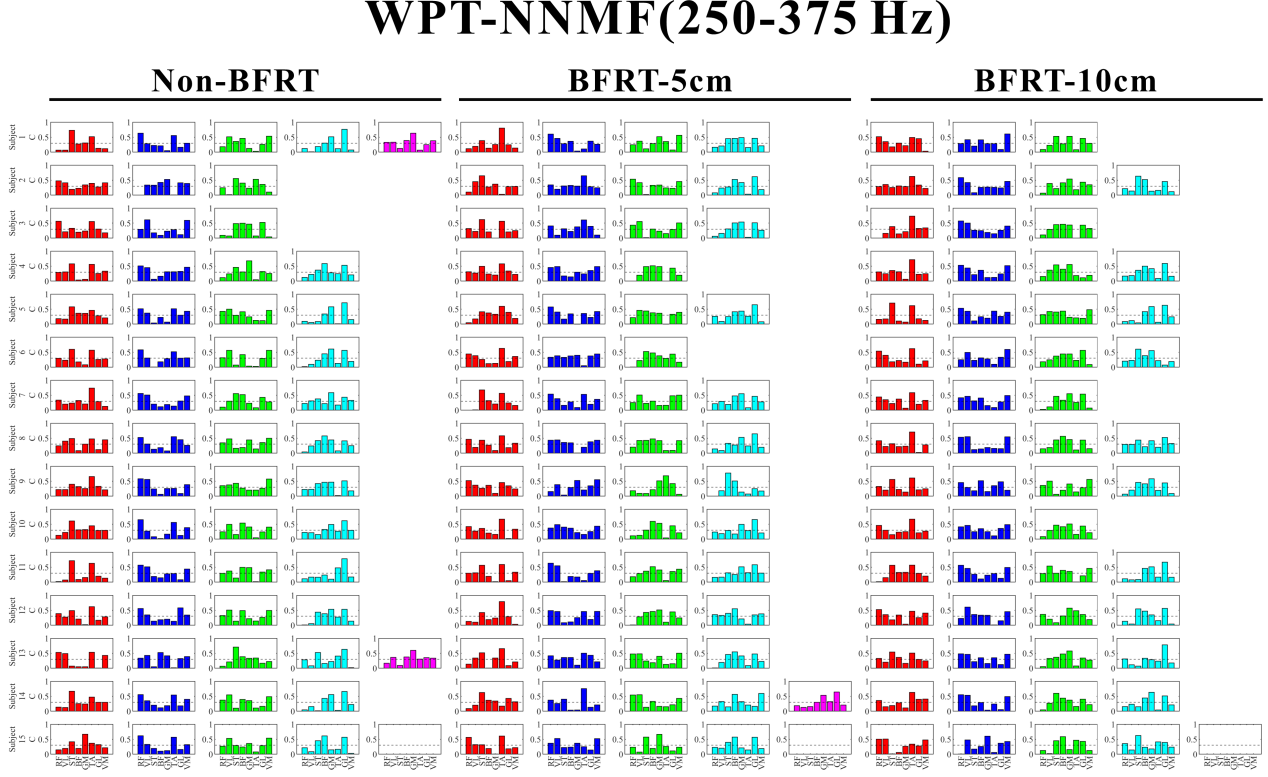

Supplement: Supplementary file 1 [file sensors-25-03154-s001.zip › Supplementary Figure S4.png]

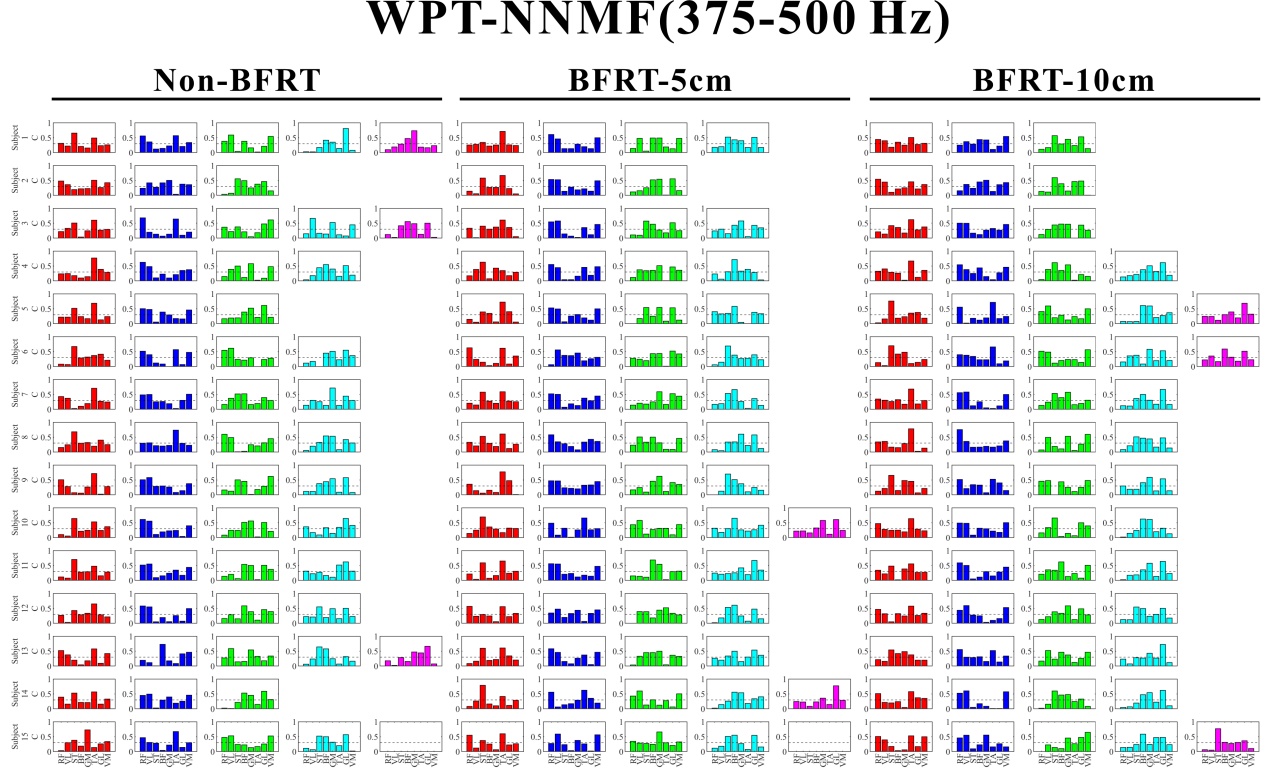

Supplement: Supplementary file 1 [file sensors-25-03154-s001.zip › Supplementary Figure S5.png]
